# Clinical significance and immune landscapes of stemness‐related and immune gene set‐based signature in oral cancer

**DOI:** 10.1002/ctm2.343

**Published:** 2021-02-19

**Authors:** Xian Lin, Xiongzhou Zheng, Baihua Yang, Jian Chen, Qin Xu, Qingwen Wang

**Affiliations:** ^1^ Department of Rheumatism and Immunology Peking University Shenzhen Hospital Shenzhen Peking University ‐ The Hong Kong University of Science and Technology Medical Center Shenzhen Guangdong People's Republic of China; ^2^ Shenzhen Key Laboratory of Immunity and Inflammatory Diseases Peking University Shenzhen Hospital Shenzhen Peking University ‐ The Hong Kong University of Science and Technology Medical Center Shenzhen Guangdong People's Republic of China; ^3^ Department of Otorhinolaryngology Xianyou County General Hospital Xianyou Fujian People's Republic of China; ^4^ Department of Radiation Oncology Fujian Cancer Hospital and Fujian Medical University Cancer Hospital Fujian Medical University Fuzhou Fujian People's Republic of China

To the Editor:

In this investigation, we constructed and validated a signature that may have clinical implications to estimate the prognosis of oral cancer patients, optimize immunotherapies for oral cancer, and identify a branch of T1‐2N0‐1 oral cancer patients suitable for adjuvant therapy. The relationship among an eight‐gene signature and clinicopathological characteristics, immune landscapes, and somatic variation profiles of oral cancer suggested the role of the signature in helping illuminate the underlying mechanisms among oral cancer stemness, immunity, and recurrence.

Oral cancer is a leading cause of morbidity and mortality and the sixth most common cancer worldwide.^1^ Although several strategies were adopted for oral cancer therapy, the survival rates of oral cancer patients have barely improved due to recurrence. Adjuvant therapy is employed after primary surgery for oral cancer patients with a high risk of recurrence based on multiple factors.^2^ Therefore, it is meaningful to identify factors for predicting oral cancer recurrence. Stemness and immunity are associated with the prognosis of human cancers. Cancer stemness is recognized as the dominant factor of cancer initiation, progression, and therapy resistance. However, whether the stemness‐ and immune‐related gene signature could serve as a predictor of prognosis and recurrence in oral cancer remains undetermined.

mRNAsi and mDNAsi have been used as effective stemness index in pan‐cancer.^3^ The distribution of stemness index and differentially expressed genes between oral cancer and normal tissues were explored (Figure S1). Oral cancer tissues presented a higher stemness compared to normal tissues by using mRNAsi and mDNAsi indices, which were correlated with the overall survival of oral cancer patients. WGCNA was then employed to reveal genes strongly correlated with oral cancer stemness and construct a gene coexpression network (Figure S2). To comprehensively estimate the relationship between stemness index and immunity in oral cancer patients, the intersection of stemness index‐ and immune‐related genes was performed and generated 86 key genes (Table S1). In addition, the enrichment analyses suggested the involvement of these genes in cancer stemness‐ and immunity‐associated signaling, including extracellular matrix, DNA replication, cell cycle, human papillomavirus infection, PI3K‐Akt signaling, and so on (Figure S3).

The LASSO and Cox regression models were applied to construct a final signature consisting of eight genes (Table S2), and the eight‐gene risk model was an unfavorable and independent prognostic factor for overall survival and recurrence‐free survival of oral cancer patients after adjusting for age, grade, TNM stage, T stage and lymphatic metastasis (Figure S4).

The risk model performed well in predicting overall survival and recurrence‐free survival of oral cancer patients from TCGA and GSE41613 (Figures 1 and 2). The association of risk score with clinicopathological characteristics is described in Tables S3 and S4. The constructed risk model predicted poor overall survival and recurrence‐free survival in different clinical subgroups of oral cancer patients (Figures S5–S7). Adjuvant therapy is not recommended for T1‐2N0‐1 oral cancer patients according to the National Comprehensive Cancer Network guideline due to a low level of evidence, while adjuvant radiation for T1‐2N1 oral cancer patients was confirmed to be beneficial.^4^ Interestingly, strong prognostic performance in T1‐2N0‐1 oral cancer patients made our identified signature more attractive for clinical translation. Moreover, the nomograms were also employed for predicting survival status and relapse status in oral cancer patients from TCGA and GSE41613 (Figure S8).

**FIGURE 1:**
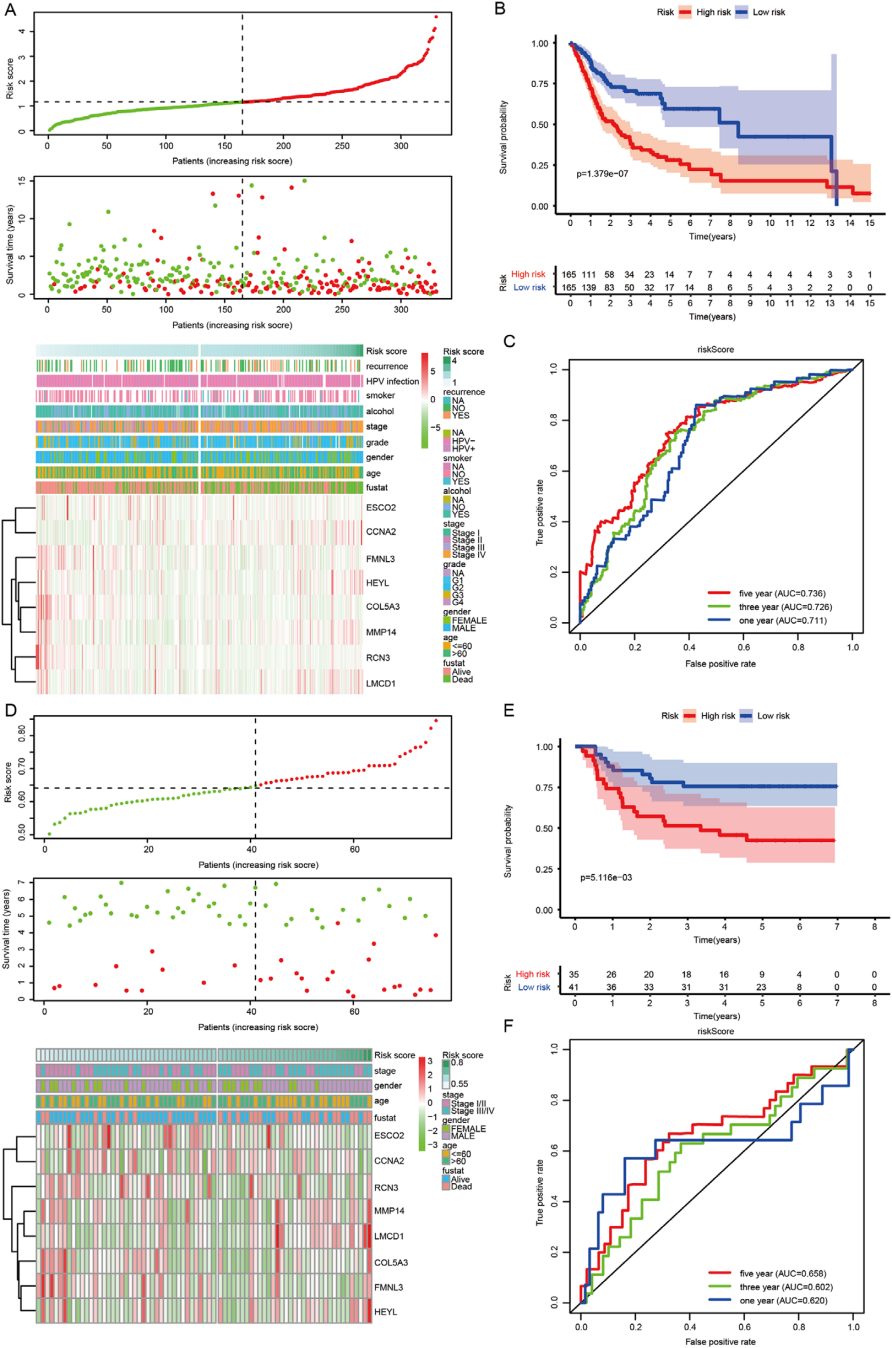
Establishment and confirmation of an eight‐gene risk model and its predictive performance for oral cancer patients. (A) The global overview of risk score, survival status, and gene expression pattern in the TCGA training cohort. (B) Kaplan–Meier curve of the correlation between risk score and overall survival of oral cancer patients in the TCGA training cohort. (C) The ROC curves show the performance of the risk model for predicting overall survival at 1, 3, and 5 years in the TCGA training cohort. (D) The global overview of risk score, survival status, and gene expression pattern in GEO validation cohort. (E) Kaplan–Meier curve of the correlation between risk score and overall survival of oral cancer patients in GEO validation cohort. (F) The ROC curves show the performance of the risk model for predicting overall survival at 1, 3, and 5 years in the GEO validation cohort. GEO: Gene Expression Omnibus; TCGA: The Cancer Genome Atlas

**FIGURE 2:**
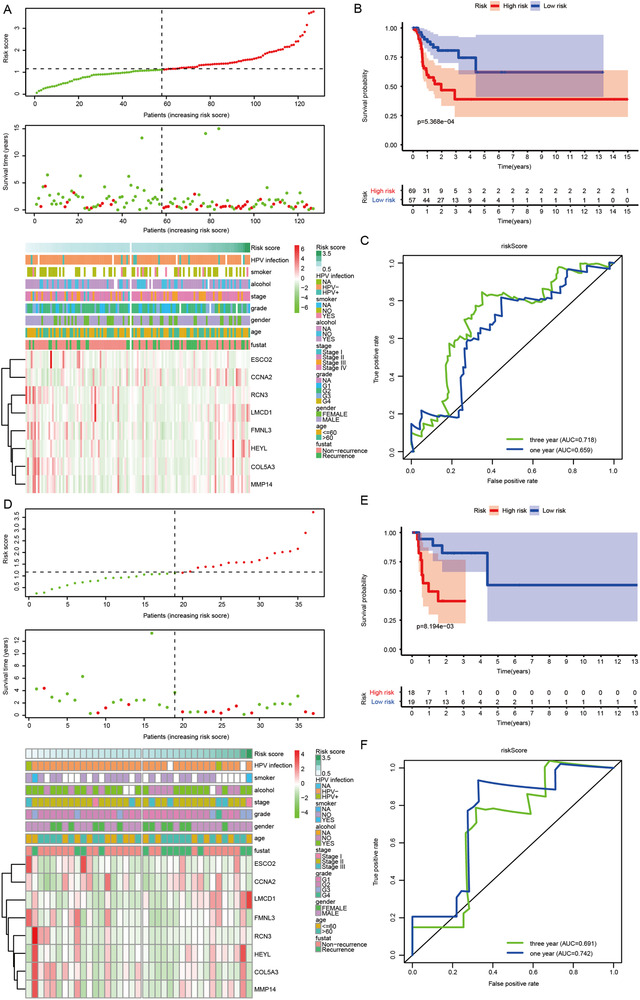
Verification of the constructed risk model in different clinical subgroups of oral cancer patients. (A) The distribution of risk score, recurrence status, and gene expression pattern in the TCGA cohort. (B) Kaplan–Meier curve of the correlation between risk score and recurrence‐free survival of oral cancer patients in the TCGA cohort. (C) The ROC curves show the performance of the risk model for predicting recurrence‐free survival at 1 and 3 years in the TCGA cohort. (D) The distribution of risk score, recurrence status, and gene expression pattern in oral cancer patients with pathologically staged T1‐2N0‐1 from the TCGA cohort. (E) Kaplan–Meier curve of the correlation between risk score and recurrence‐free survival of oral cancer patients with pathologically staged T1‐2N0‐1 from TCGA cohort. (F) The ROC curves show the performance of the risk model for predicting recurrence‐free survival at 1 and 3 years in oral cancer patients with pathologically staged T1‐2N0‐1 from the TCGA cohort. TCGA: The Cancer Genome Atlas

In order to clarify the underlying mechanism connecting the immune‐related gene signature and the risks of mortality and relapse, several immune profile‐relevant analytical strategies were adopted. First, we investigated the link between the eight‐gene signature and six immune subtypes.^5^ Second, ssGSEA was utilized to make the links more interpretable based on the newly generated 61 immune gene sets. Next, estimate algorithms^6^ were applied to calculate stormal score, immune score, estimate score, and tumor purity. Last, the analyses were extended to incorporate 30 immune checkpoint molecules, including the B7‐CD28 family, the TNF superfamily, and several other immune checkpoint members.^7^ In both TCGA and GEO cohorts, high‐risk score calculated by the eight‐gene signature presented a consistent relationship with immune depletion, immune suppression, and resistance to immune checkpoint blockade, leading to a poor prognosis and a high possibility of recurrence (Figures 3 and 4A–K). Furthermore, the enrichment analyses verified the involvement of risk score in modulating oral cancer immunity in both TCGA and GEO cohorts (Figure S9).

**FIGURE 3:**
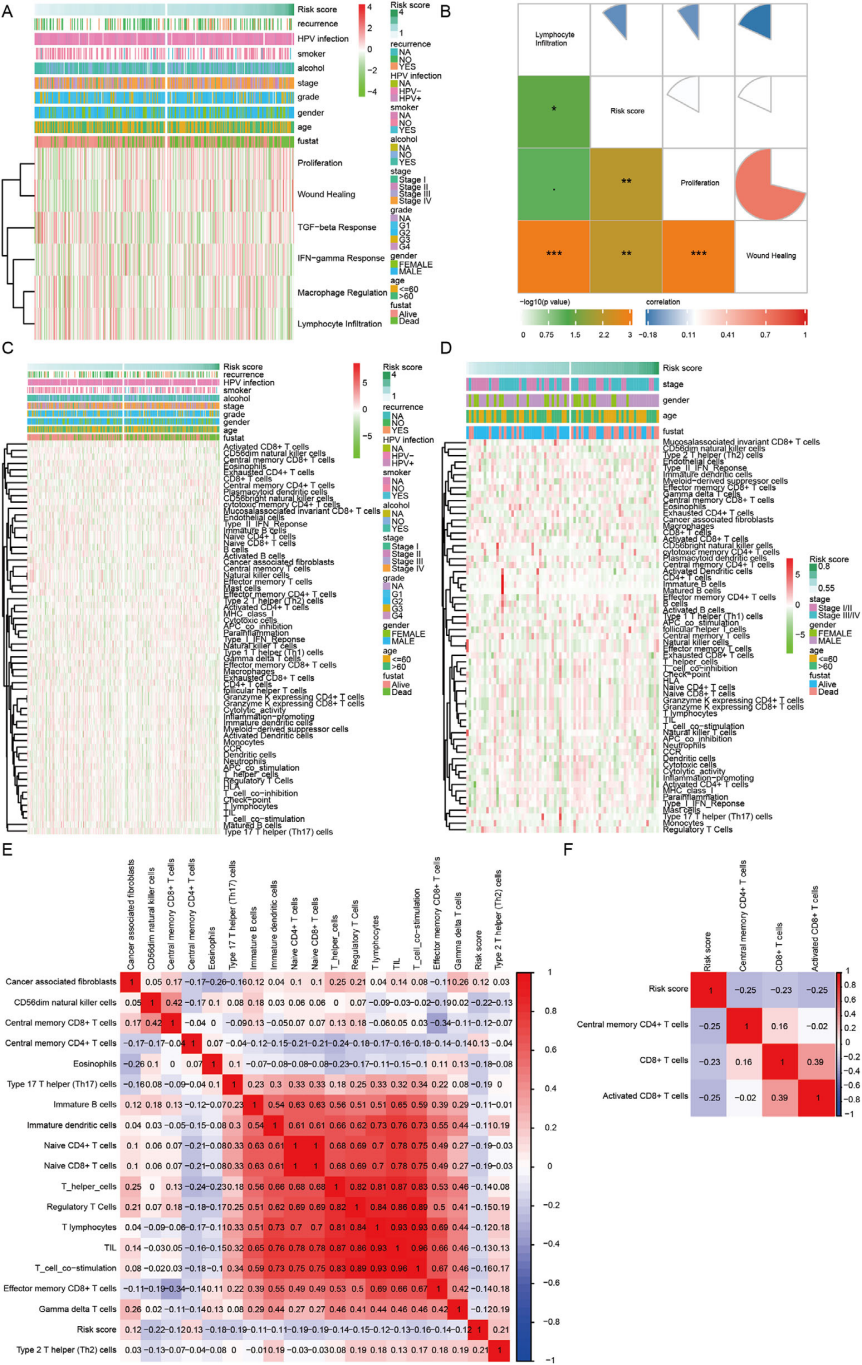
The connection between risk score and clusters of immune subtypes, immune cells infiltration, and immune‐related processes in oral cancer. (A) The link between risk score and six clusters of immune subtypes in the TCGA training cohort. (B) The correlogram displaying the link between risk score and three immune subtypes of proliferation, wound healing, and lymphocyte infiltration in the TCGA training cohort. (C and D) Estimated immune cells and immune‐related processes in patients with low‐ and high‐risk scores in TCGA training and GEO validation cohorts, respectively. (E and F) The correlograms were used to show relationships between risk score and immune cells and immune‐related processes in TCGA training and GEO validation cohorts, respectively. GEO: Gene Expression Omnibus; TCGA: The Cancer Genome Atlas

**FIGURE 4:**
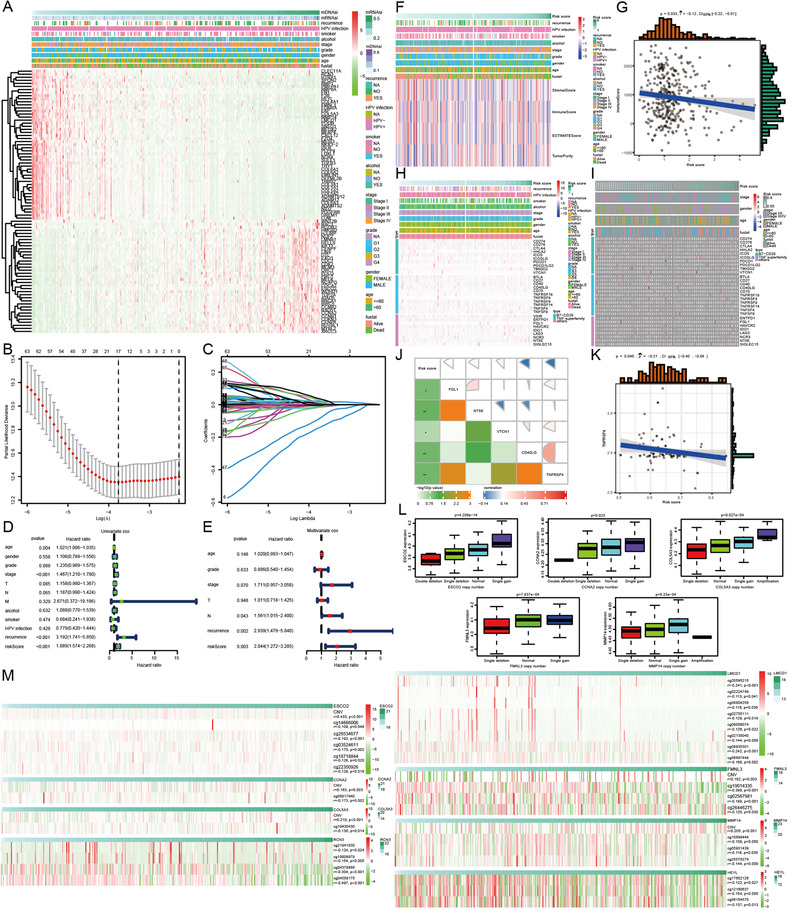
The connection among oral cancer patients’ survival, immune score and immune checkpoint molecules, and risk score calculated by the eight‐gene signature whose expression was affected by DNA copy number variation and DNA methylation in oral cancer. (A) The expression patterns of 86 immune genes significantly correlated with stemness index. (B) Tuning parameter selection using 10‐fold cross‐validation in LASSO regression analysis. (C) Coefficient profiles of the prominent prognostic genes in LASSO regression analysis. (D) Forest plot displaying the role of clinicopathological parameters and risk score for predicting oral cancer patients’ survival in the univariate Cox proportional hazard regression model. (E) Forest plot displaying the role of clinicopathological parameters and risk score for predicting oral cancer patients’ survival in the multivariate Cox proportional hazard regression model. (F) The link between risk score and scores calculated by estimate algorithms in the TCGA training cohort. (G) The correlogram displaying the negative relationship between risk score and immunescore in the TCGA training cohort. (H and I) The expression profiles of 30 immune checkpoint molecules in patients with low‐ and high‐risk scores in TCGA training and GEO validation cohorts, respectively. (J and K) The correlograms of the relationship between risk score and immune checkpoint molecules in TCGA training and GEO validation cohorts, respectively. (L) Differential expression of five key genes in SIBS in oral cancer patients with a different type of DNA copy number variation. (M) The relationship between DNA copy number variation and DNA methylation of key genes in SIBS and the expression of their corresponding genes. GEO: Gene Expression Omnibus; LASSO: least absolute shrinkage and selection operator; SIBS: stemness‐related and immune gene set‐based signature; TCGA: The Cancer Genome Atlas

Studies reported the link between immune infiltration and alterations in the tumoral genome.^8^ However, the difference in genomic changes in the top 10 mutated genes between oral cancer patients with high‐ and low‐risk scores was not significant, and the differentially genomic changes only existed in a small proportion of oral cancer patients from the TCGA cohort with a percentage lower than 5.5% (Figure S10). As expected, no significant association between risk score and tumor mutation burden was elucidated (Table S5), suggesting that there are other factors affecting oral cancer immunity independently of somatic alterations.

DNA copy number variation and hypomethylation were associated with immune escape signatures and immunotherapeutic resistance.^9^ Furthermore, DNA copy number variation and hypomethylation also influenced the specific gene expression.^10^ In the present investigation, we consistently revealed that DNA copy number variation and hypomethylation regulated the expression of the eight key genes in our established risk model, further modulating the stemness, immunity, and recurrence of oral cancer (Figure 4L,M).

Collectively, our work portrays an eight‐gene signature that contributes to clarifying the link among stemness, immunity, and prognosis of oral cancer patients (Figure S11). Oral cancer patients with high‐risk scores are not suitable for immune checkpoint blockade therapy, and intensified treatment strategies for these patients should be considered. Moreover, the signature can serve as an efficient and accurate tool for medical decision making and individualized treatment, especially for the selection of T1‐2N0‐1 oral cancer patients who may benefit from adjuvant therapy, thus effectively reducing oral cancer recurrence. The conclusions were based on bioinformatic analyses, and further experiments are required to verify these findings.

## CONFLICT OF INTEREST

The authors declare that there is no conflict of interest.

## DATA AVAILABILITY STATEMENT

The data that support the findings of this study are openly available in the TCGA database (https://www.cancer.gov/tcga) and the GEO database (http://www.ncbi.nlm.nih.gov/geo/).

## FUNDING INFORMATION

National Natural Science Foundation of China, Grant Number: 81974253; Nature Science Foundation of Guangdong Province, Grant Number: 2019A1515011112; Key Project of Basic Research of Shenzhen Science and Technology Innovation Commission, Grant Number: JCYJ20200109140203849; Shenzhen Sanming Project of Shenzhen Municipal Health Commission, Grant Number: SZSM201612009

## Supporting information

Supporting Information

Supporting Information

Supporting Information
