# Supplementary material for: Clinical significance and immune landscapes of stemness‐related and immune gene set‐based signature in oral cancer
Source: Clin Transl Med. 2021 Feb 19;11(2):e343. doi: 10.1002/ctm2.343 (PMC7893806; doi:10.1002/ctm2.343)
Supplement: Supplementary file 1 — Supporting Information [file CTM2-11-e343-s001.pdf]

LETTER TO EDITOR

Clinical significance and immune landscapes of stemness-related and immune gene-set-based signature in oral cancer

Xian Lin, et al

Supplemental materials

Methods

Data sources

mRNA expression profiles, genomic data, and clinicopathological annotations of oral cancer and normal tissues were downloaded from the TCGA database (https://www.cancer.gov/tcga). Microarray and clinicopathological data of oral cancer were downloaded from the GSE41613 in the GEO database (http://www.ncbi.nlm.nih.gov/geo/). Therein, only patients who died of oral cancer were extracted from GSE41613. Totally, 330 oral cancer patients in the TCGA database and 76 oral cancer patients in the GEO database were assigned to the training cohort and validation cohort, respectively. Immune signatures were collected from previous studies, [1, 2] and 61 gene sets were generated. Moreover, immune-related genes were gathered from the GSEA Website (https://www.gsea-msigdb.org/gsea/index.jsp) and ImmPort Database (https://www.immport.org/home).

Weighted gene co-expression network analysis (WGCNA)

The limma package in R was applied to identified differentially expressed genes (DEGs) in oral cancer tissues versus normal tissues in the TCGA cohort. The criteria

for DEGs selection was $P < 0.05$, false discovery rate (FDR) < 0.05 and |log2 fold change| > 1. The WGCNA package in R was applied to reveal genes strongly correlated with oral cancer stemness and to develop a gene co-expression network targeting DEGs. mRNA expression-based stemness index (mRNAsi) and DNA methylation-based stemness index (mDNAsi) were obtained from a previous study [3] and were chosen as the representative traits to explore the cancer stem cells (CSCs) properties-associated genes and modules. To evaluate the significance of the identified modules, the gene significance (GS) values were calculated to analyse the interaction between gene expression and sample characteristics. The pheatmap and limma packages in R were utilized to portray heatmap and volcano plot, respectively.

Functional annotation and pathway enrichment analysis

The clusterProfiler package in R and gene set enrichment analysis (GSEA) software were applied to perform GO functional annotation and KEGG pathway analyses. The enriched biological processes (BP), cellular component (CC), and molecular function (MF) were acquired for GO enrichment analyses. Statistical significance was assigned as q value < 0.05. The bar-plot, bubble-plot, circos, chord, and mutiGSEA were portrayed with R packages to display the top terms.

Cox regression analyses for SIBS generation

The least absolute shrinkage and selection operator (LASSO) Cox regression analyses were carried out to select the appropriate variables for modeling, followed by conducting the Cox proportional hazards regression analyses to optimize the risk model. Finally, a risk score was calculated by considering optimized Cox regression

coefficients and mRNA expression based on a linear combination. Oral cancer patients were divided as the high- or low-risk group through ranking the calculated risk score. The Kaplan–Meier curves and log-rank tests were adopted to assess overall survival (OS) and recurrence-free survival (RFS) between the high- and low-risk group in TCGA and GEO cohorts. The Cox proportional hazards regression analyses were applied for screening independent prognostic factors. The time-dependent receiver operating characteristic (ROC) curves were generated through running the survivalROC package in R. The rms package in R was used to establish a prognosis nomogram for predicting the individual possibility of survival and recurrence status for oral cancer patients.

Immune cell infiltration and immune-related processes analyses

The abundance of immune cell infiltration and immune-related processes in oral cancer patients with high- and low- risk score was estimated by single sample GSEA (ssGSEA) based on Gene Sets Variation Analysis (GSVA). The ssGSEA method is suitable for sample level enrichment analysis according to the absolute expression levels of marker genes in a single sample.

Somatic mutation profiling

The maftools package in R was applied to summarize, analyse and display mutation annotation, and tumour mutation burden (TMB) was calculated. The summary of mutation annotation summarizes the number of variants as a barplot and variant types as a boxplot. The oncoplots, also known as waterfall plots, were also delineated to represent somatic mutation. The somaticInteractions function was adopted to detect

co-occurring or mutually exclusive set of genes. Forest plots were generated to visualize the difference of somatic mutation between different groups.

Statistical analysis

The data analyses and the generation of figures were carried out with R software version 4.0.2 (https://www.r-project.org). The Wilcoxon rank-sum tests were applied to investigate the difference of mRNAsi and mDNAsi scores between oral cancer and normal tissues, as well as differential expression of 5 key genes in SIBS in oral cancer patients with a different type of copy number variation. The Chi-square tests were utilized to examine the correlations between risk scores and clinicopathological characteristics. The correlations among calculated scores, gene expressions and genomic alterations were measured using Spearman's rank correlation tests. Univariate and multivariate analyses were carried out with a Cox proportional hazard regression model. *P* value of < 0.05 was considered statistically significant.

References

1. Zuo S, Wei M, Wang S, Dong J, Wei J. Pan-Cancer Analysis of Immune Cell Infiltration Identifies a Prognostic Immune-Cell Characteristic Score (ICCS) in Lung Adenocarcinoma. Front Immunol 2020;11:1218.

2. He Y, Jiang Z, Chen C, Wang X. Classification of triple-negative breast cancers based on Immunogenomic profiling. J Exp Clin Cancer Res 2018;37:327.

3. Malta TM, Sokolov A, Gentles AJ, Burzykowski T, Poisson L, Weinstein JN, et al. Machine Learning Identifies Stemness Features Associated with Oncogenic Dedifferentiation. Cell 2018;173:338-354.e315.

Supplemental figures

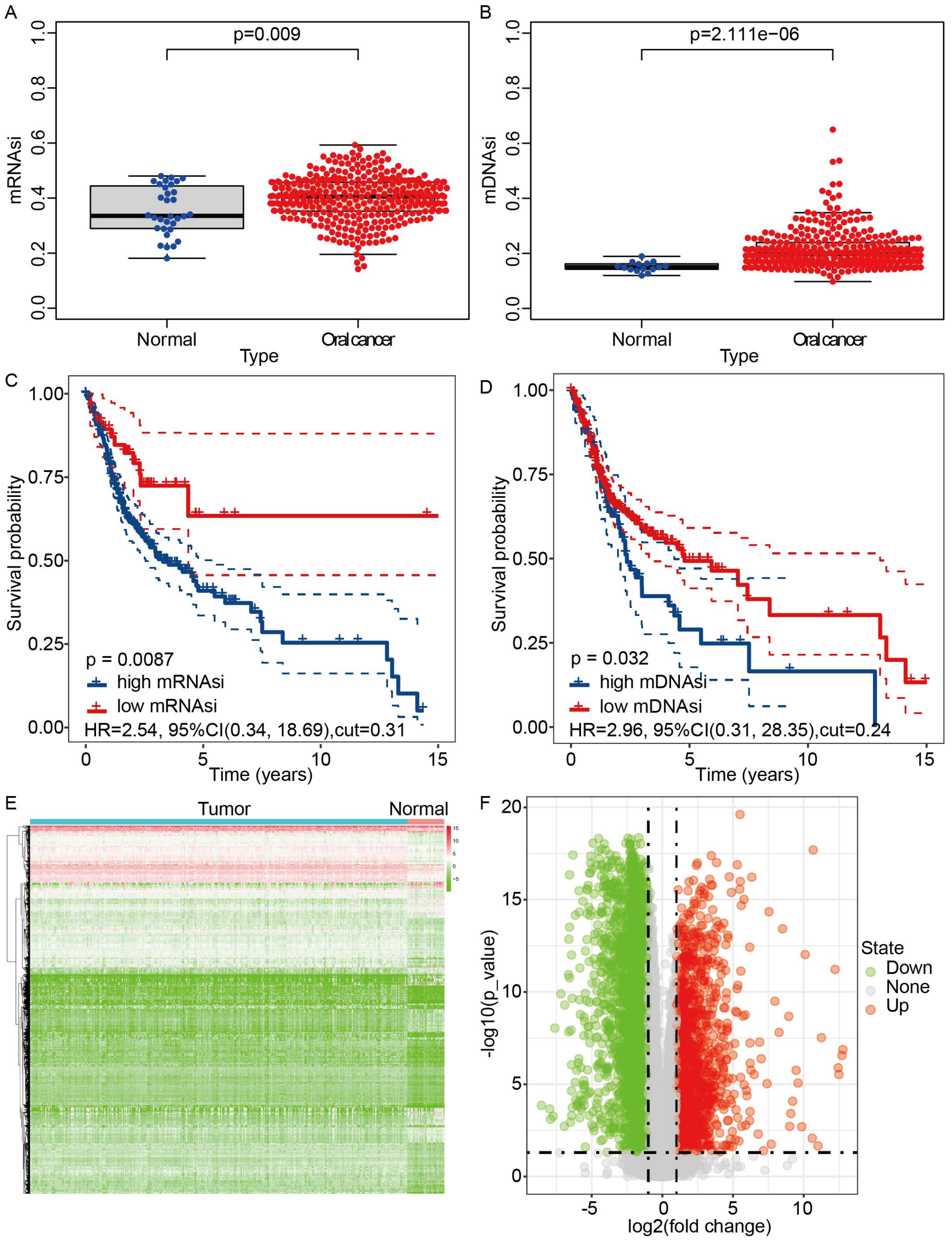

Figure S1. The stemness index and DEGs between oral cancer and normal tissues based on the TCGA database. (A) Differential mRNAsi in oral cancer and normal tissues. (B) Differential mDNAsi in oral cancer and normal tissues. (C) Survival curves of mRNAsi in oral cancer patients in the TCGA database. (D) Survival curves

of mDNAsi in oral cancer patients in the TCGA database. (E) Heatmap of differentially expressed genes between oral cancer and normal tissues. (F) Volcano plot of differentially expressed genes between oral cancer and normal tissues. DEGs: differentially expressed genes; TCGA: The Cancer Genome Atlas; mRNAsi: mRNA expression-based stemness index; mDNAsi: DNA methylation-based stemness index.

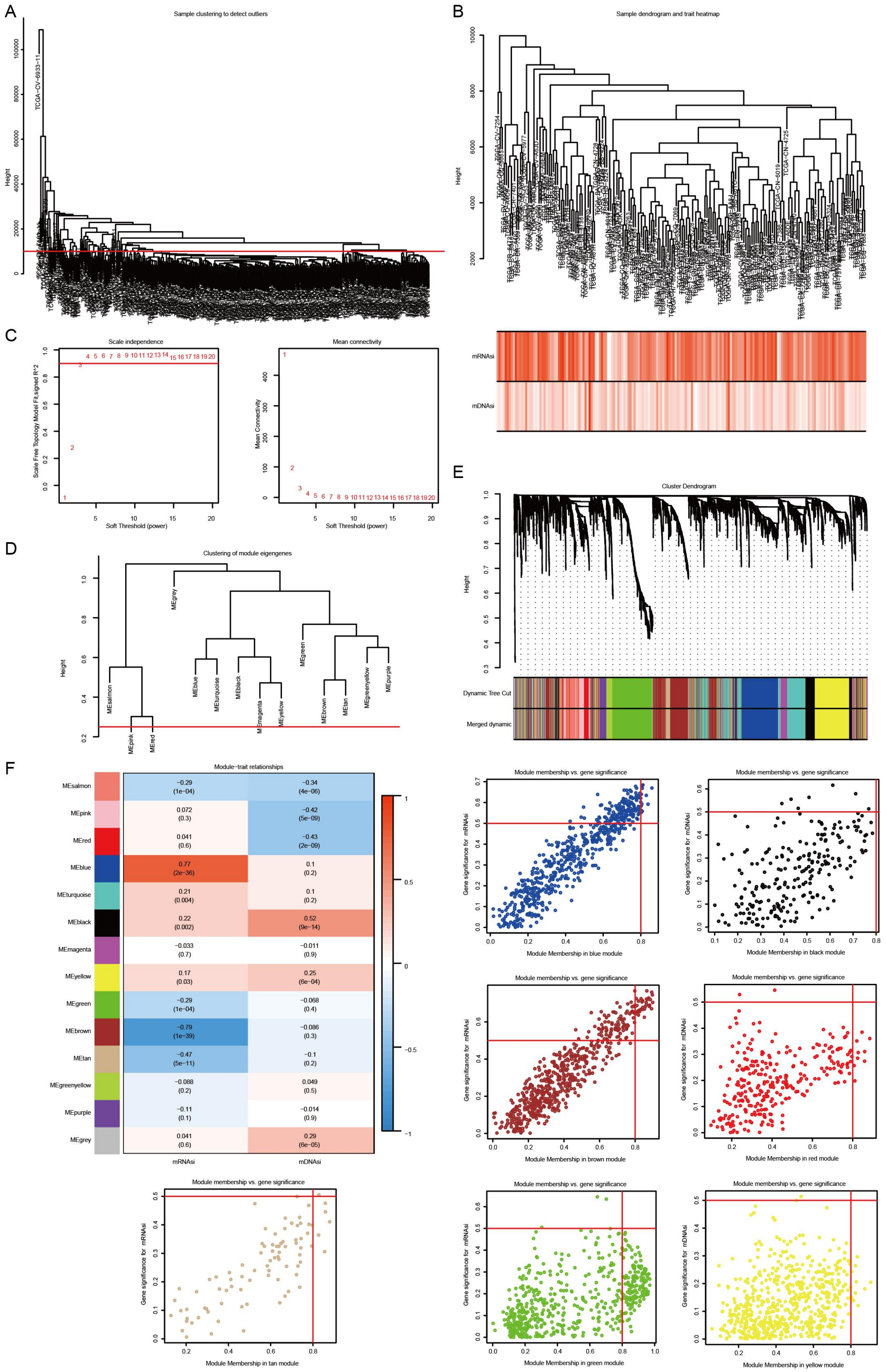

Figure S2. Weighted gene co-expression network analysis. (A) The normal or

missing data over the red line were removed for the outlier elimination. (B) The dendrogram showing the distribution of each oral cancer sample, and the heatmap displaying the distribution of the mRNAsi and mDNAsi in oral cancer. (C) The power value = 4 was selected according to the scale independence and mean connectivity. (D) A GeneTree containing 14 different gene modules was established based on the power value. (E) The 14 different gene modules were delineated as the branches of the cluster dendrogram. Each leaf represented a gene on the cluster dendrogram. Each module with a unique color indicated a cluster of co-related genes. The heatmap exhibiting different gene modules with co-related genes, and the modules with high similarity were merged by calculating the module similarity. (F) The heatmap shows the significant differences and associations between mRNAsi or mDNAsi and the gene module. The upper row and the brackets in gene modules represented the correlation coefficient and *P* value, respectively. The scatter plot exhibits module eigengenes in the blue, black, brown, red, green, yellow, and tan modules linked to mRNAsi or mDNAsi. Each circle represents a gene, and the circles in the upper stand for the key genes in the modules. mRNAsi: mRNA expression-based stemness index; mDNAsi: DNA methylation-based stemness index.

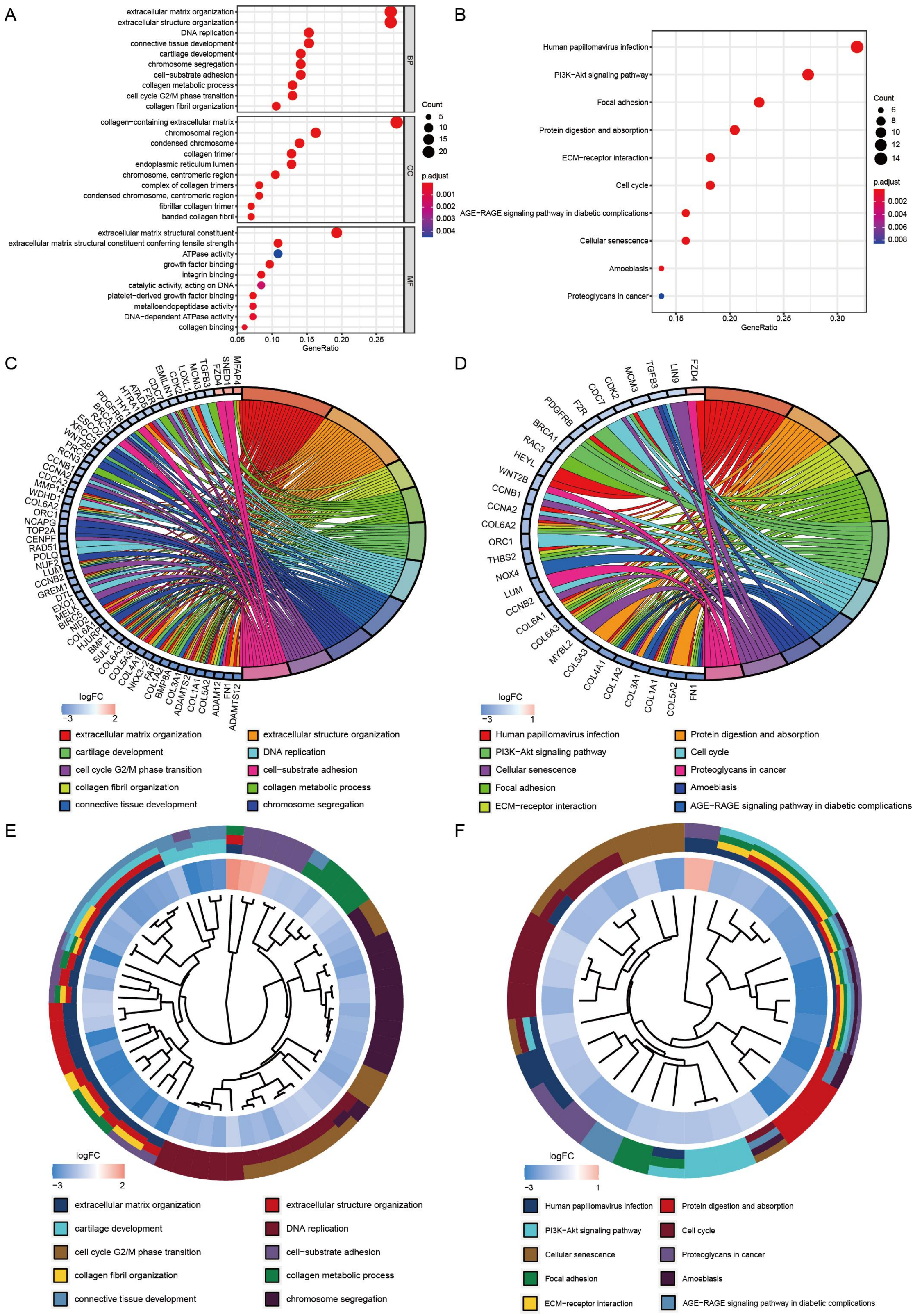

Figure S3. Gene set enrichment analysis for the key genes. (A and B) Bubble-plot shows the top terms of the enriched GO sets and KEGG pathways involved in cancer stemness- and immunity-associated signaling, including extracellular matrix, DNA

replication, cell cycle, Human papillomavirus infection, PI3K-Akt signaling, and so on. Count: Number of genes associated with the enriched GO or KEGG pathway. BP, biological process; CC, cell component; MF, molecular function. (C-F) Circos and chord show the top terms of the enriched GO sets and KEGG pathways with specific enriched genes and clusters of these specific genes.

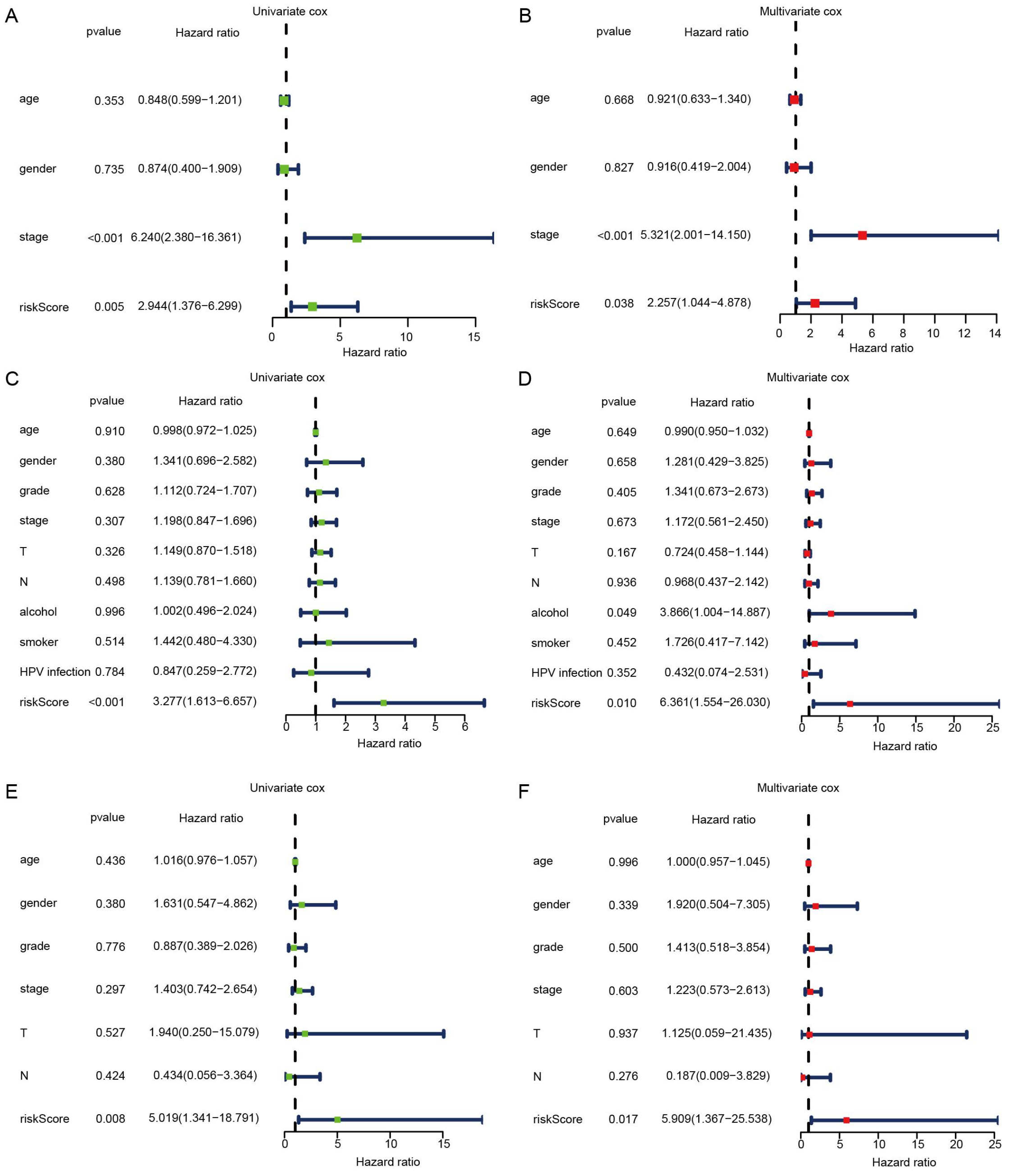

Figure S4. Univariate and multivariate Cox regression analyses of risk scores and clinicopathological characteristics with overall survival and recurrence-free survival. (A and B) Forest plot displaying the role of clinicopathological parameters and risk score for predicting overall survival of oral cancer patients from GEO training cohort in the Cox proportional hazard regression model and multivariate Cox proportional hazard regression model, respectively. (C and D) Forest plot displaying

the role of clinicopathological parameters and risk score for predicting recurrence-free survival of oral cancer patients with available recurrence status from TCGA cohort in the Cox proportional hazard regression model and multivariate Cox proportional hazard regression model, respectively. (E and F) Forest plot displaying the role of clinicopathological parameters and risk score for predicting recurrence-free survival of oral cancer patients with pathologically staged T1-2N0-1 from TCGA cohort in the Cox proportional hazard regression model and multivariate Cox proportional hazard regression model, respectively. GEO: Gene Expression Omnibus; TCGA: The Cancer Genome Atlas.

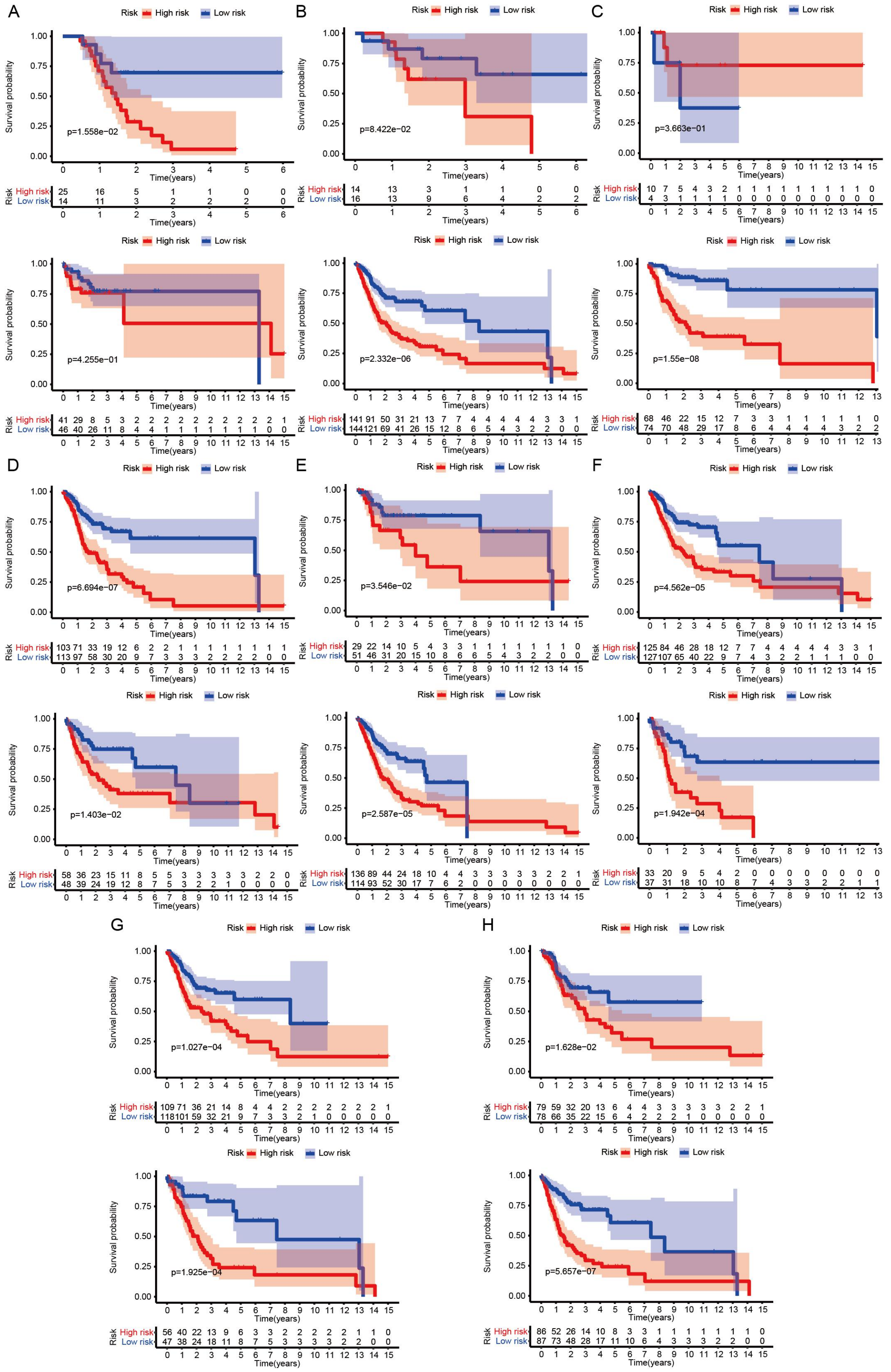

Figure S5. Survival analysis of oral patients with high- or low- risk score in the

TCGA training cohort. Kaplan-Meier curves of overall patient survival in recurrence and no recurrence (A), HPV infection and non-HPV infection (B), smoker and non-smoker (C), drinking and non-drinking (D), stage I-II and stage III-IV (E), histology grade 1-2 and histology grade 3-4 (F), male and female (G), younger (age $\leq$ 60 years) and older (age $>$ 60 years) (H) subgroups from TCGA training cohort based on risk scores. TCGA: The Cancer Genome Atlas; HPV: Human papillomavirus.

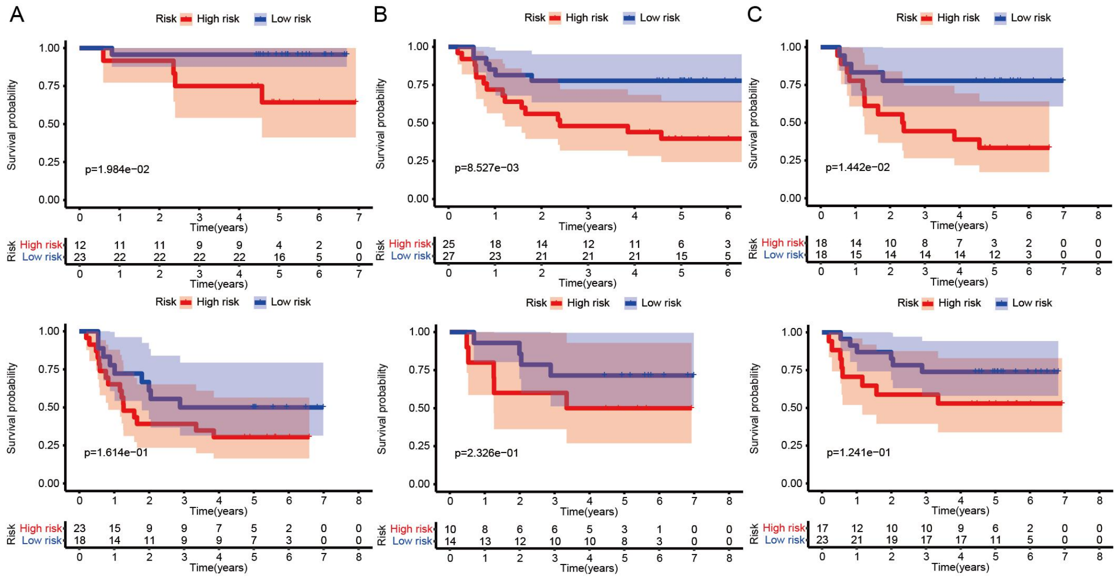

Figure S6. Survival analysis of oral patients stratified by stage, gender, and age in the GEO validation cohort. Kaplan-Meier curves of overall patient survival in stage I-II and stage III-IV (A), male and female (B), younger (age ≤ 60 years) and older (age > 60 years) (C) subgroups from GEO validation cohort based on risk scores. GEO: Gene Expression Omnibus.

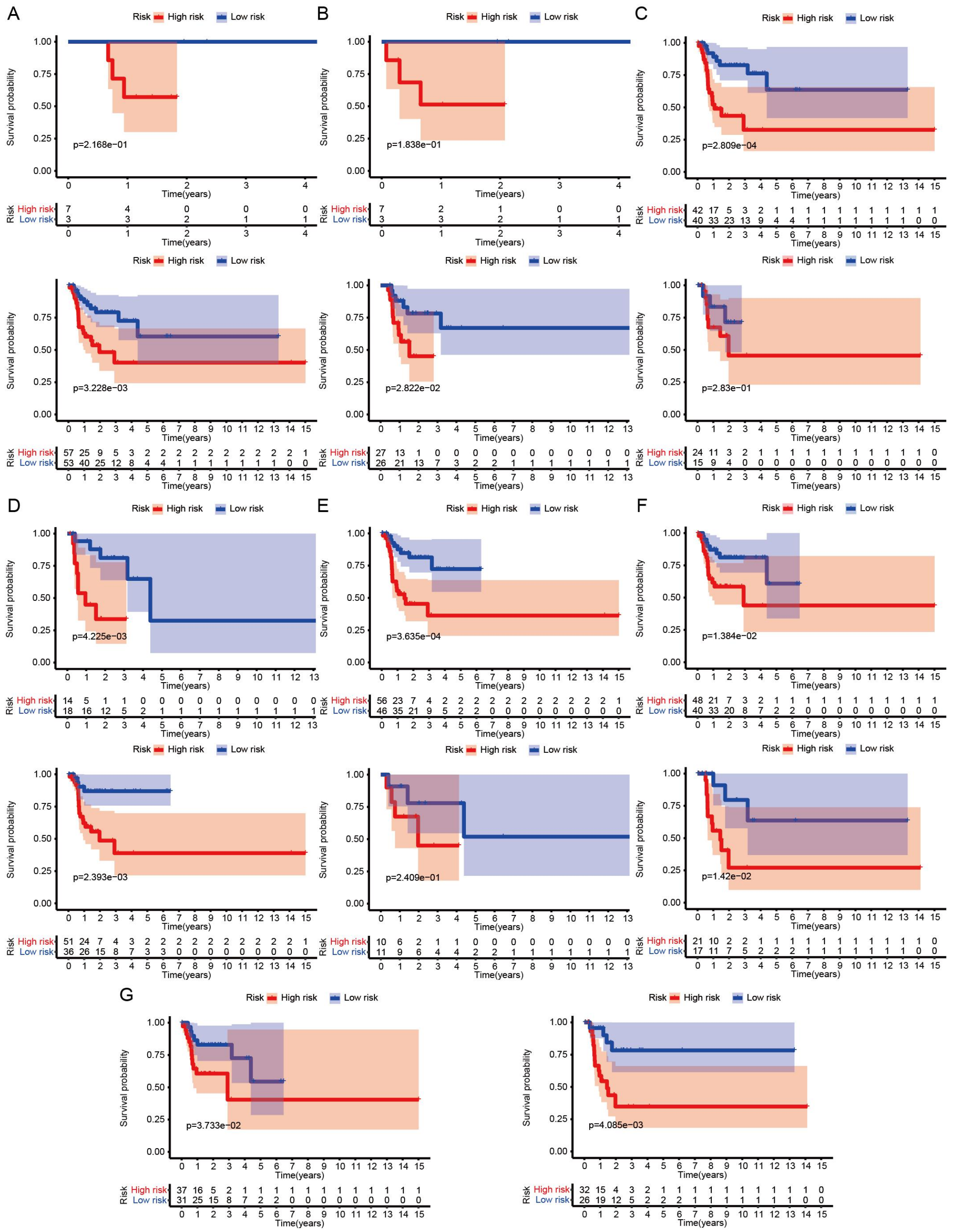

Figure S7. Validation of the prognostic performance of stemness- and immune-related gene signature stratified by HPV infection status, smoking history, drinking history, stage, histology grade, gender and age in the TCGA cohort. Kaplan-Meier curves of recurrence-free survival in HPV infection and non-HPV infection (A), smoker and non-smoker (B), drinking and non-drinking (C),

stage I-II and stage III-IV (D), histology grade 1-2 and histology grade 3-4 (E), male and female (F), younger (age $\leq$ 60 years) and older (age > 60 years) (G) patients based on risk score in the TCGA cohort. HPV: Human papillomavirus; TCGA: The Cancer Genome Atlas.

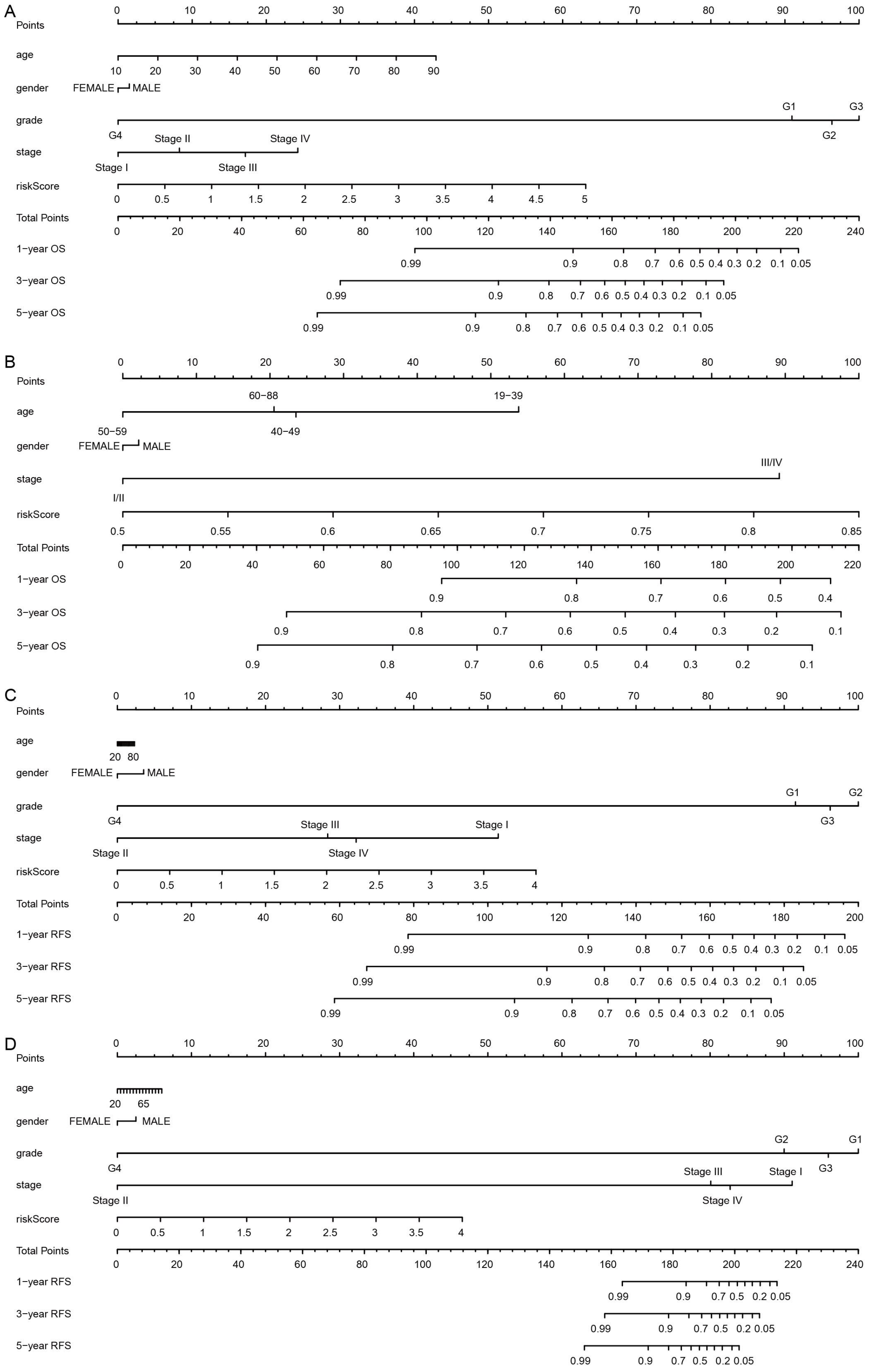

Figure S8. Construction of prognostic nomograms to predict the possibility of

survival and recurrence status for oral cancer patients. (A) A prognostic nomogram on the basis of risk score and clinical parameters for predicting survival status in TCGA training cohort. (B) A prognostic nomogram on the basis of risk score and clinical parameters for predicting survival status in GEO validation cohort. (C) A prognostic nomogram on the basis of risk score and clinical parameters for predicting relapse status in oral cancer patients with available recurrence status from the TCGA cohort. (D) A prognostic nomogram on the basis of risk score and clinical parameters for predicting relapse status in oral cancer patients with pathologically staged T1-2N0-1 from TCGA cohort. TCGA: The Cancer Genome Atlas; GEO: Gene Expression Omnibus.

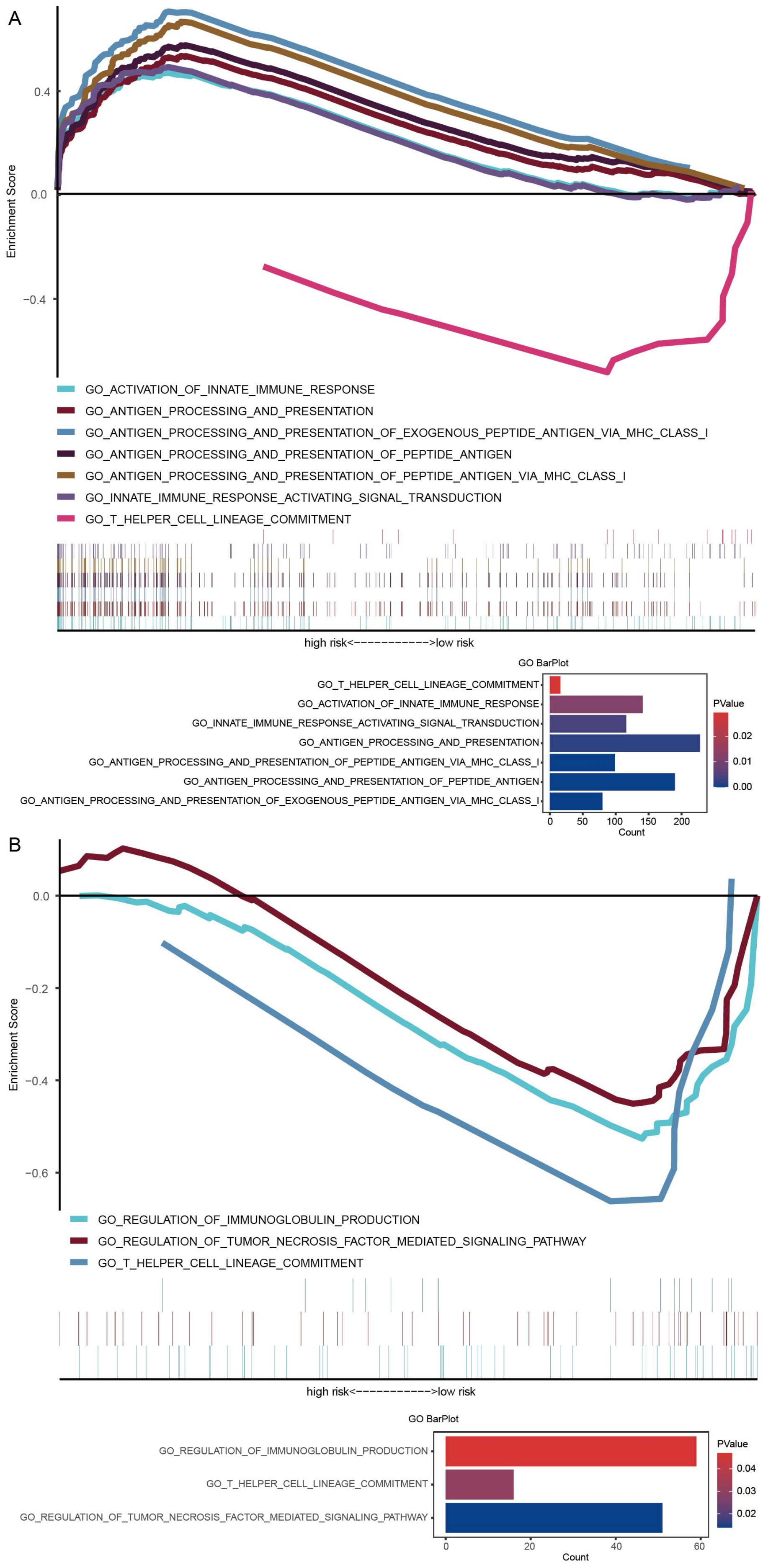
A
Enrichment Score
0.4
0.0
−0.4
GO_ACTIVATION_OF_INNATE_IMMUNE_RESPONSE
GO_ANTIGEN_PROCESSING_AND_PRESENTATION
GO_ANTIGEN_PROCESSING_AND_PRESENTATION_OF_EXOGENOUS_PEPTIDE_ANTIGEN_VIA_MHC_CLASS_I
GO_ANTIGEN_PROCESSING_AND_PRESENTATION_OF_PEPTIDE_ANTIGEN
GO_ANTIGEN_PROCESSING_AND_PRESENTATION_OF_PEPTIDE_ANTIGEN_VIA_MHC_CLASS_I
GO_INNATE_IMMUNE_RESPONSE_ACTIVATING_SIGNAL_TRANSDUCTION
GO_T_HELPER_CELL_LINEAGE_COMMITMENT
high risk<------------>low risk
GO BarPlot
GO_T_HELPER_CELL_LINEAGE_COMMITMENT
GO_ACTIVATION_OF_INNATE_IMMUNE_RESPONSE
GO_INNATE_IMMUNE_RESPONSE_ACTIVATING_SIGNAL_TRANSDUCTION
GO_ANTIGEN_PROCESSING_AND_PRESENTATION
GO_ANTIGEN_PROCESSING_AND_PRESENTATION_OF_PEPTIDE_ANTIGEN_VIA_MHC_CLASS_I
GO_ANTIGEN_PROCESSING_AND_PRESENTATION_OF_PEPTIDE_ANTIGEN
GO_ANTIGEN_PROCESSING_AND_PRESENTATION_OF_EXOGENOUS_PEPTIDE_ANTIGEN_VIA_MHC_CLASS_I
0
50
100
150
200
Count
PValue
0.02
0.01
0.00
B
Enrichment Score
0.0
−0.2
−0.4
−0.6
GO_REGULATION_OF_IMMUNOGLOBULIN_PRODUCTION
GO_REGULATION_OF_TUMOR_NECROSIS_FACTOR_MEDIATED_SIGNALING_PATHWAY
GO_T_HELPER_CELL_LINEAGE_COMMITMENT
high risk<------------>low risk
GO BarPlot
GO_REGULATION_OF_IMMUNOGLOBULIN_PRODUCTION
GO_T_HELPER_CELL_LINEAGE_COMMITMENT
GO_REGULATION_OF_TUMOR_NECROSIS_FACTOR_MEDIATED_SIGNALING_PATHWAY
0
20
40
60
Count
PValue
0.04
0.03
0.02

Figure S9. Gene set enrichment analysis identifies the participation of SIBS in oral cancer immunity. (A) MutiGSEA and barplot show the enriched GO sets between high- and low-risk score groups in the TCGA training cohort. (B) MutiGSEA and barplot show the enriched GO sets between high- and low-risk score groups in the GEO validation cohort. SIBS: stemness-related and immune gene-set-based signature; TCGA: The Cancer Genome Atlas; GEO: Gene Expression Omnibus.

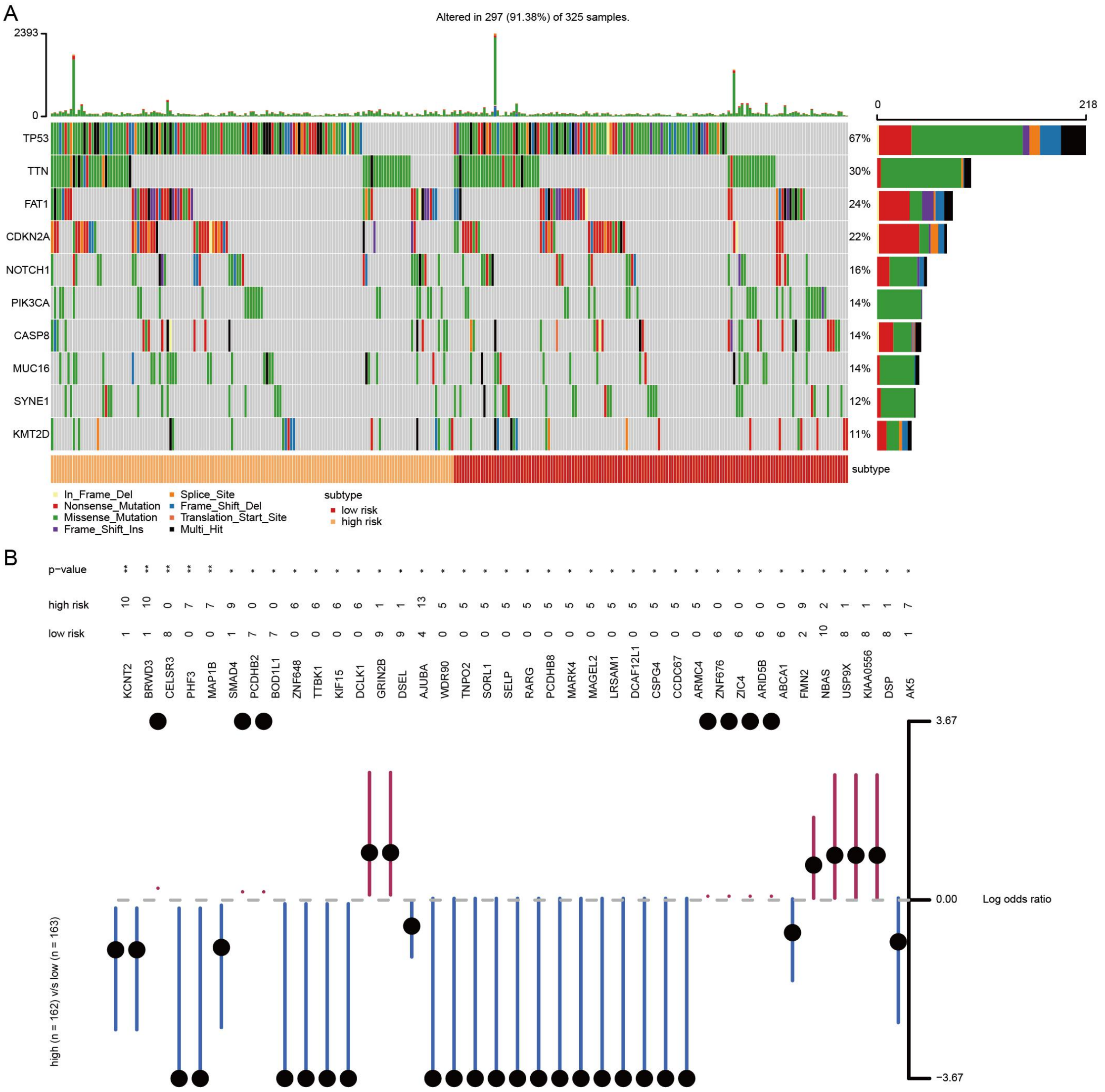

Figure S10. Somatic variation profiles of SIBS in oral cancer from TCGA training cohort. (A) Top 10 somatic alterations in oral cancer with high- and low-risk scores. (B) Differential somatic alterations in oral cancer with high- and low-risk scores. SIBS: stemness-related and immune gene-set-based signature; TCGA: The Cancer Genome Atlas.

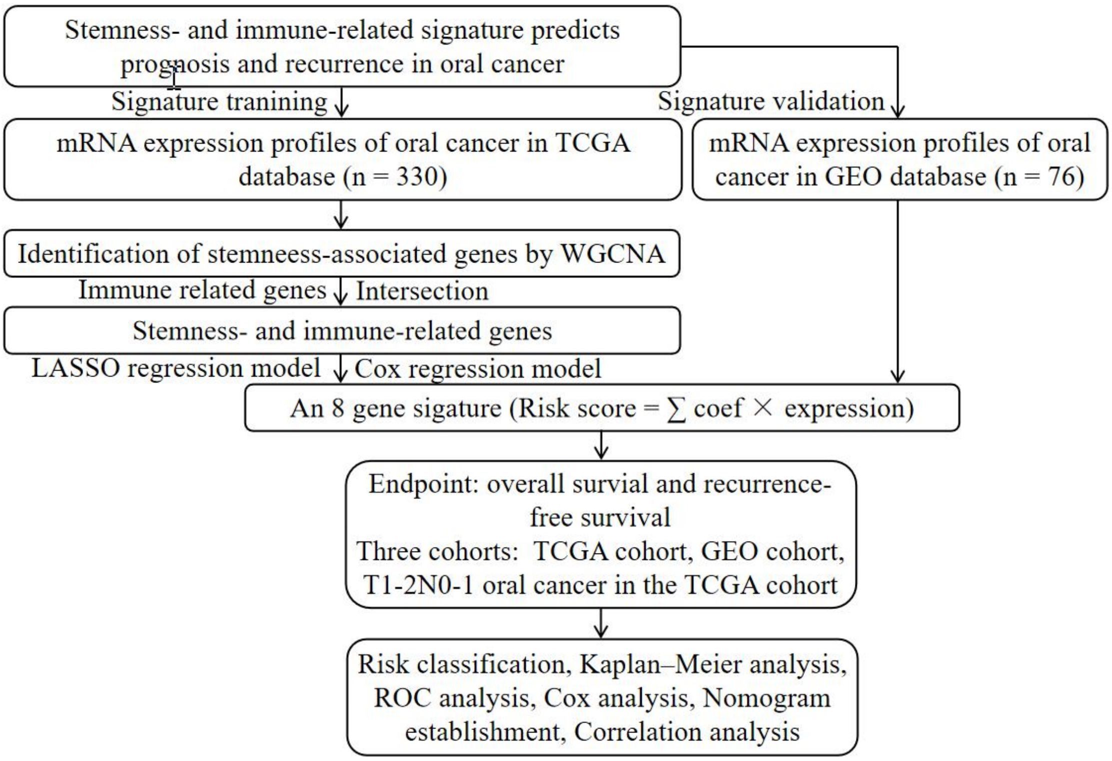

Figure S11. The stemness- and immune-related gene signature generation and validation pipeline.

Supplemental tables

Table S2. The 8 genes in prognostic model in TCGA cohort

id	coef	HR	HR.95L	HR.95H	*P* value
ESCO2	-0.21769201	0.804373143	0.670310926	0.965247808	0.019273557
CCNA2	0.027623791	1.028008866	1.010686876	1.045627734	0.001442553
COL5A3	-0.038646458	0.962090789	0.938897558	0.985856953	0.001909108
RCN3	-0.018894895	0.981282495	0.967620799	0.995137078	0.008255386
LMCD1	0.168062045	1.183010009	1.056133247	1.325128893	0.003690149
FMNL3	-0.210446774	0.810222179	0.694329757	0.945458512	0.007538003
MMP14	0.004838883	1.00485061	1.001806317	1.007904153	0.001773719
HEYL	0.123638473	1.131606691	1.033037019	1.239581622	0.007837858

Table S3. Correlations between risk score and the clinicopathological features of TCGA training cohort

Characteristics	n	Risk score		*P* value
		Low	High	
Age (years)				
≤Median	157	78 (49.7%)	79 (50.3%)	0.912
＞Median	173	87 (50.3%)	86 (49.7%)	
Gender				
Female	103	47 (45.6%)	56 (54.4%)	0.285
Male	227	118 (52.0%)	109 (48.0%)	
Drinking				
No	106	48 (45.3%)	58 (54.7%)	0.236
Yes	216	113 (52.3%)	103 (47.7%)	
Smoking				
No	142	74 (52.1%)	68 (47.9%)	0.159
Yes	14	4 (28.6%)	10 (71.4%)	
Stage				
Stage I-II	80	51 (63.7%)	29 (36.3%)	0.005
Stage III-IV	250	114 (45.6%)	136 (54.4%)	
HPV infection				
Negative	285	144 (50.5%)	141 (49.5%)	0.770
Positive	30	16 (53.3%)	14 (46.7%)	
Histology grade				
G1-2	252	127 (50.4%)	125 (49.6%)	0.716
G3-4	70	37 (52.9%)	33 (47.1%)	
Recurrence				
No	87	46 (52.9%)	41 (47.1%)	0.078
Yes	39	14 (35.9%)	25 (64.1%)	

Table S4. Correlations between risk score and the clinicopathological features of GEO testing cohort

Characteristics	n	Risk score		*P* value
		Low	High	
Age (years)				
≤Median	36	18 (50.0%)	18 (50.0%)	0.512
＞Median	40	23 (57.5%)	17 (42.5%)	
Gender				
Female	24	14 (58.3%)	10 (41.7%)	0.602
Male	52	27 (51.9%)	25 (48.1%)	
Stage				
Stage I-II	35	23 (65.7%)	12 (34.3%)	0.057
Stage III-IV	41	18 (43.9%)	23 (56.1%)	
